# Lung Metastases in Endometrial Carcinoma: A Deadly Twist

**DOI:** 10.7759/cureus.61109

**Published:** 2024-05-26

**Authors:** Souvik Sarkar, Ulhas Jadhav, Pankaj Wagh, Jay Bhanushali, Amit Toshniwal, Arman Sindhu, Bingu Shiv Kiran Reddy

**Affiliations:** 1 Respiratory Medicine, Datta Meghe Institute of Higher Education and Research, Wardha, IND

**Keywords:** endometrial biopsy, hypoxic respiratory failure, endometrial carcinoma, adenocarcinoma of endometrium, lung metastasis

## Abstract

This case report describes the clinical course of a 73-year-old postmenopausal female presenting with a persistent cough, breathlessness, and hypertension. Upon examination, she exhibited signs of respiratory distress, prompting transfer to the intensive care unit (ICU) where type 1 respiratory failure was diagnosed. Chest imaging revealed bilateral lung opacities, leading to a diagnosis of lung metastasis. Subsequent screening investigations unveiled endometrial carcinoma with atypical respiratory symptoms, highlighting the importance of thorough evaluation. Despite prompt management and biopsy confirmation, the patient's condition rapidly deteriorated, underscoring the aggressive nature of metastatic endometrial carcinoma. This case underscores the necessity of considering atypical presentations and timely intervention in managing such malignancies.

## Introduction

In 2020, endometrial cancer ranked as the second most prevalent gynaecological malignancy globally and stood as the fourth primary cause of death attributed to malignancies in the field of gynaecology [1]. Endometrial cancer tends to occur more frequently in developed nations; India, being a developing nation, experiences a comparatively lower incidence rate in comparison to other developed countries [2]. In this article, we present a case of endometrial carcinoma in which the patient initially presented with respiratory symptoms, notably breathlessness and cough, without any abdominal or gynaecological complaints. However, the patient was later diagnosed with metastatic endometrial carcinoma. Sadly, our patient succumbed to the advanced lung lesions before receiving any treatment or surgery for the primary endometrial mass. This case report highlights the uncommon presentation of extensive disease associated with endometrial carcinoma and underscores the importance of paying attention to even the slightest complaints experienced by individuals.

## Case presentation

**Figure 1:**
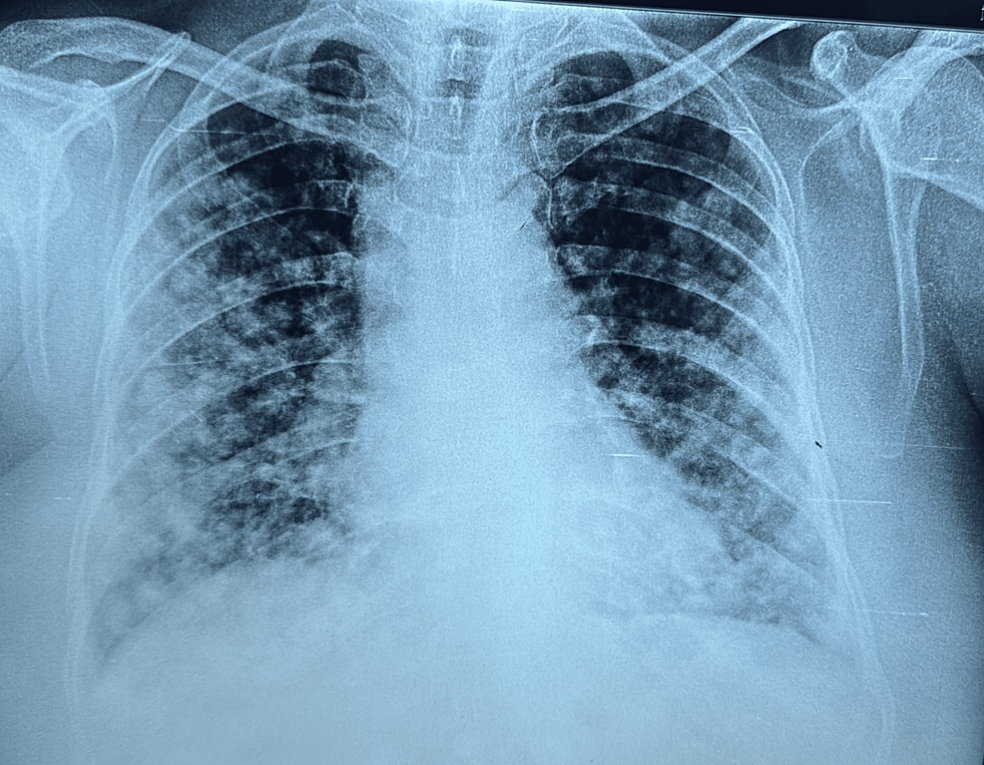
Chest X-ray postero-anterior view showing multiple bilateral variable-sized nodules throughout the lung parenchymal areas with patchy inhomogeneous opacities predominantly in the lower zones.

A 73-year-old obese, postmenopausal female presented with a persistent cough and expectoration lasting for three months, along with worsening breathlessness, now even occurring at rest over the same duration. The patient was non-diabetic and had a history of hypertension for the past five years but had been irregular with her medications. Upon examination, she appeared dyspnoeic and agitated but remained conscious, oriented, and responsive to commands. Her body mass index was 33.2, pulse rate was 118 beats per minute, with a respiratory rate of 44 breaths per minute, and blood pressure reading was 150 systolic by 90 diastolic mm Hg. She had an oxygen saturation of 89% on ambient air and oedema in both lower limbs, and bilateral crackles were heard predominantly in the basal areas of the thorax on respiratory auscultation. Consequently, the patient was promptly transferred to the intensive care unit (ICU), where an arterial blood gas analysis was done which revealed type 1 respiratory failure (partial pressure of oxygen (PaO2) of 45). The rest of her lab investigations were all within normal limits. A chest X-ray revealed bilateral multiple rounded opacities of varying sizes throughout the lung fields, accompanied by diffuse patchy opacities (Figure 1).

**Figure 2:**
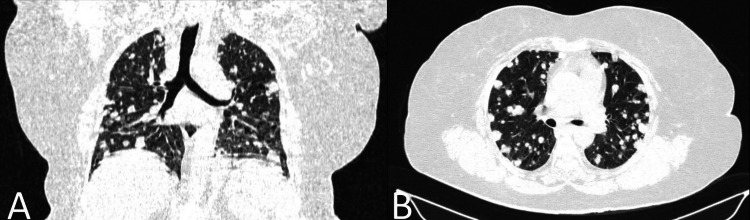
(A) Coronal section of HRCT-thorax revealing multiple variable-sized (2-20 mm diameter) soft tissue density nodules dispersed bilaterally throughout the lung parenchyma. (B) Axial section of HRCT-thorax displaying analogous widespread nodules indicative of metastatic lung disease. HRCT-thorax: high-resolution computed tomography of the thorax

Initiating immediate management, the patient received noninvasive ventilation, nebulizations, steroids, diuretics, and intravenous antibiotics. Within 24 hours, her chest auscultation improved, and oxygen saturation stabilized at 96% with 2 L of oxygen support via nasal prongs. Subsequently, high-resolution computed tomography of the thorax (HRCT-thorax) was performed, showing multiple, variable-sized (size 2-20 mm diameter) round to oval soft tissue nodules scattered throughout the lung parenchyma bilaterally, indicating lung metastasis, prompting further investigation (Figure 2).

**Figure 3:**
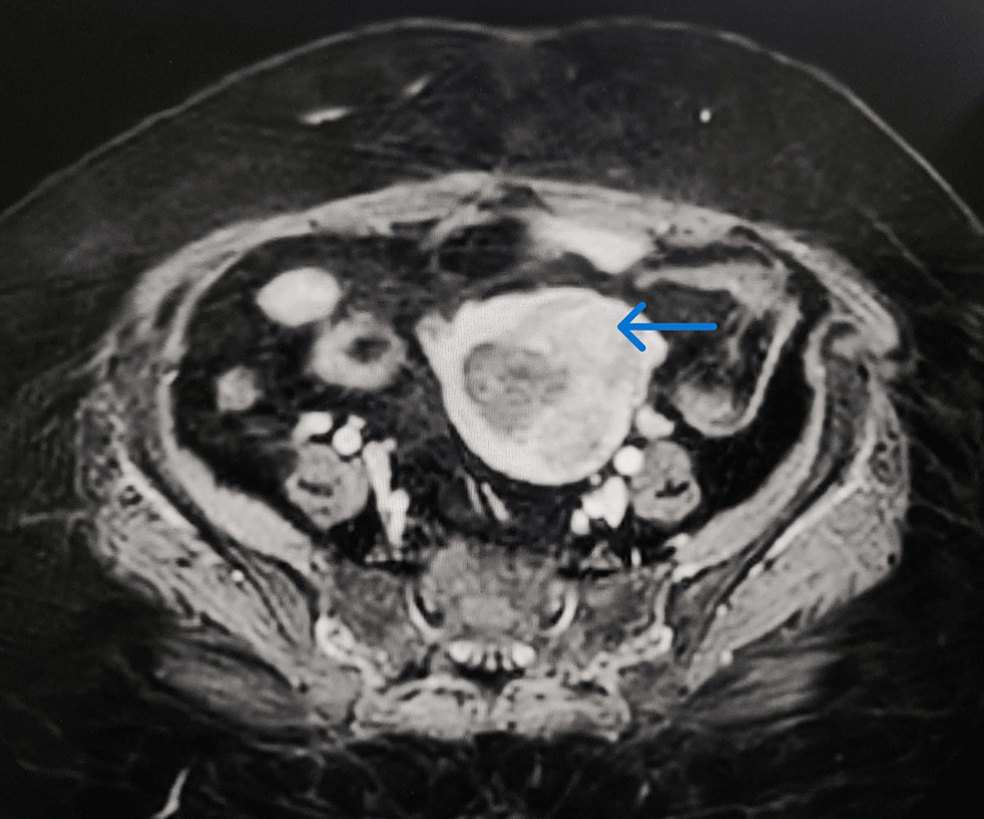
MRI-angiography confirmed the presence of a bulky uterus with heterogeneously enhancing lesion in the endometrial cavity with loss of endometrial-myometrial junction at the fundus and upper cornu on the left side. The lesion measured approximately 5.4 × 4.2 × 5.3 cm in side with a thickened endometrium of approximately 3 cm. MRI-angiography: magnetic resonance imaging with angiography

Screening ultrasound of the abdomen and pelvis revealed a bulky uterus with a thickened endometrium. Upon further questioning, the patient then disclosed having sporadic vaginal bleeding occurring postmenopause, a symptom she had largely disregarded or neglected. She had her menarche at 12 years of age and menopause at 55 years of age and had three children. Additionally, she reported experiencing lower abdominal pain accompanied by cramps quite often. On per vaginal examination, a firm, lobulated mass could be felt without any active bleeding or pain on touching. Magnetic resonance imaging with angiography (MRI-angiography) confirmed the presence of a bulky uterus with a heterogeneously enhancing lesion (5.4 × 4.2 × 5.3 cm) in the endometrial cavity with loss of endometrial-myometrial junction at the fundus and upper cornu on the left side. A few subcentimetric pre-sacral and left external iliac lymph nodes were also noted, with normal bladder, ovaries, and fallopian tubes (Figure 3).

**Figure 4:**
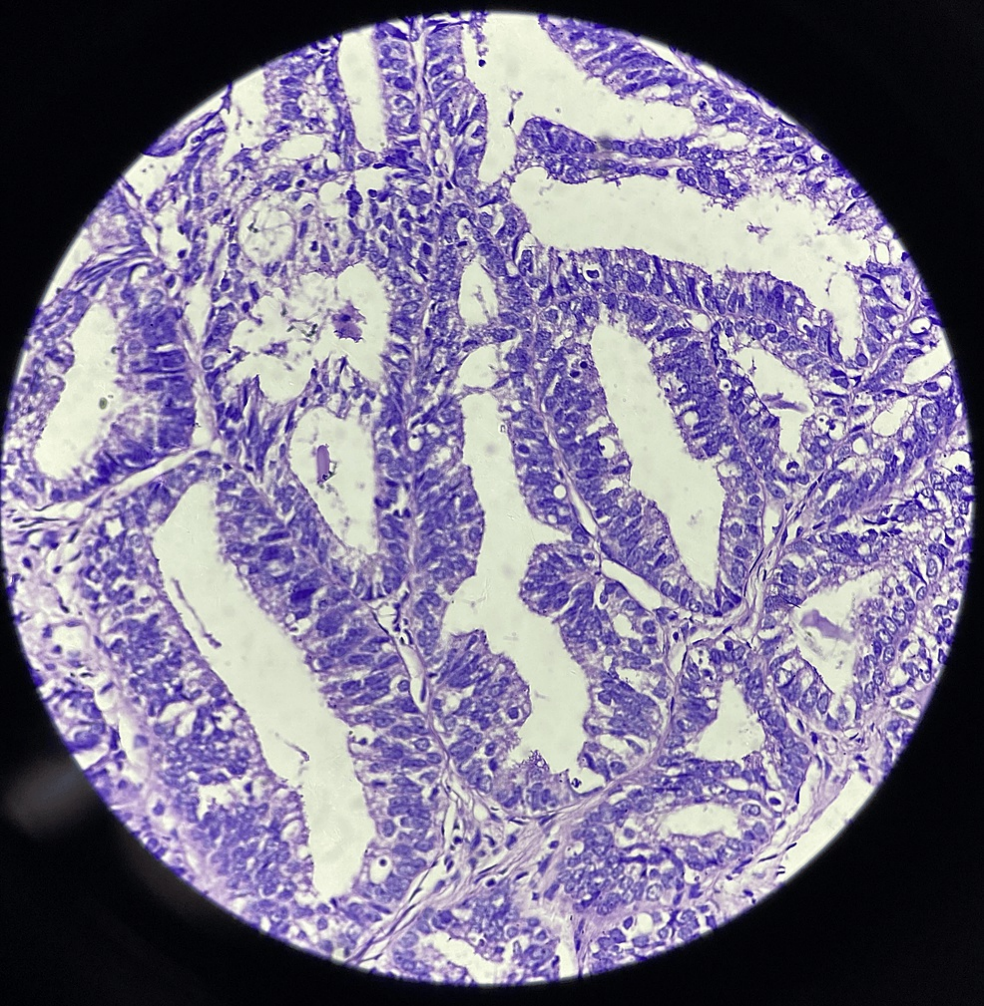
The histopathological slide section showing endometrial glands showing pleomorphic hyperchromatic nuclei with irregular nuclear membrane, irregularly clumped chromatin, abnormal mitosis, and loss of polarity of cells, features indicative of adenocarcinoma of the endometrium (well-differentiated type).

Following consultation with gynaecologists, an endometrial biopsy was performed, and tissue samples were sent for histopathological examination. The biopsy showed endometrial glands showing pleomorphic hyperchromatic nuclei with irregular nuclear membrane, irregularly clumped chromatin, abnormal mitosis, and loss of polarity on histopathology, features indicative of adenocarcinoma of the endometrium, specifically of the well-differentiated type (Figure 4).

Tragically, despite medical efforts, the patient's condition rapidly deteriorated during her hospitalization. Within a day of diagnosis, she experienced sudden worsening of breathlessness and hypoxemia, ultimately succumbing to cardiac arrest.

## Discussion

The precise cause of endometrial cancer remains elusive, yet prolonged estrogen exposure emerges as a key contributing factor, particularly in women with early initiation of menstruation and late occurrence of menopause. Some other risk factors include nulliparity, obesity, and certain medical conditions such as hypertension and diabetes, as well as the use of estrogen-only hormone replacement therapy (HRT). Genetic predispositions like Lynch syndrome and Cowden syndrome also influence susceptibility. Endometrial cancer predominantly affects postmenopausal women, with the most incidence in the elderly age group of 55-65 years [2]. In our patient, the etiology of the endometrial carcinoma still remains unclear; although she was obese, had a delayed menopause, and had a history of hypertension, she did not have any significant history of prolonged hormonal exposure and could not be tested for any specific hereditary genetic disorders. In early stages, endometrial carcinoma boasts an outstanding clinical outcome; conversely, the course for the metastatic version of endometrial carcinoma is bleak. Notably, the lungs emerge as the most prevalent site for distant metastasis in endometrial carcinoma [3]. Lung manifestations of endometrial carcinoma often exhibit multiple bilateral nodules, with the potential for a solitary nodule resembling primary lung cancer; cavitation, primarily in squamous cell types, may occur. Additionally, rare pulmonary presentations encompass lymphangitic carcinomatosis and endobronchial tumor spread, with infrequent pleural involvement like effusions, nodularity, and thickening [4]. For stage I or II endometrial cancer, the standard treatment typically involves performing a total abdominal hysterectomy and bilateral salpingo-oophorectomy, with the option of pelvic and/or para-aortic lymphadenectomy [5]. In the case of advanced endometrial cancer, chemotherapy stands as the preferred treatment for individuals facing recurrence or metastatic disease. Initial chemotherapy commonly involves a combination of medications, such as cisplatin/doxorubicin/paclitaxel, or cisplatin/doxorubicin, or carboplatin/paclitaxel, with subsequent treatment utilizing a single medication if tolerated [6]. Our patient had complaints predominantly because of respiratory metastatic lesions, which caused her to seek medical attention. Any sort of intervention or treatment for the lung lesions, as well as the primary endometrial lesion, could not be done due to the delayed presentation and the advanced stage of the aggressive tumor.

## Conclusions

This case highlights the aggressive nature of metastatic endometrial carcinoma, stressing the need for careful evaluation of respiratory symptoms in postmenopausal women. Despite the absence of typical gynaecological complaints, metastatic endometrial carcinoma should be considered, and hence, a thorough gynaecological history should be taken in all postmenopausal females with respiratory complaints and lung nodules on radiographic investigations. The rapid decline and fatal outcome underscore the aggressive nature of this disease. Early recognition of risk factors and clinical features is vital for timely diagnosis and management, especially in identifying metastatic disease. Further research is needed to understand the mechanisms of metastasis and improve outcomes for affected patients.

## References

[1] Sung H, Ferlay J, Siegel RL, Laversanne M, Soerjomataram I, Jemal A, Bray F (2021). Global Cancer Statistics 2020: GLOBOCAN estimates of incidence and mortality worldwide for 36 cancers in 185 countries. CA Cancer J Clin.

[2] Agarwal S, Melgandi W, Sonkar DR, Ansari FA, Arora S, Rathi AK, Singh K (2023). Epidemiological characteristics of endometrial cancer patients treated at a tertiary health center in National Capital Territory of India. J Cancer Res Ther.

[3] Li J, Sun L, Zhang Y, Cai S (2019). Patterns of distant metastases in patients with endometrial carcinoma: a SEER population-based analysis. J Clin Oncol.

[4] Kurra V, Krajewski KM, Jagannathan J, Giardino A, Berlin S, Ramaiya N (2013). Typical and atypical metastatic sites of recurrent endometrial carcinoma. Cancer Imaging.

[5] Creutzberg CL, van Putten WL, Koper PC (2000). Surgery and postoperative radiotherapy versus surgery alone for patients with stage-1 endometrial carcinoma: multicentre randomised trial. Lancet.

[6] Dizon DS (2010). Treatment options for advanced endometrial carcinoma. Gynecol Oncol.

